# A comprehensive database of crystal-bearing magmas for the calibration of a rheological model

**DOI:** 10.1038/s41597-022-01363-w

**Published:** 2022-05-30

**Authors:** Alessandro Frontoni, Antonio Costa, Alessandro Vona, Claudia Romano

**Affiliations:** 1 Dipartimento di Scienze, sez. Geologia, Università degli Studi Roma Tre, L.go San Leonardo Murialdo 1, 00146 Rome, Italy; 2 Istituto Nazionale di Geofisica e Vulcanologia, Sezione di Bologna, Via Donato Creti, 12, 40128 Bologna, Italy

**Keywords:** Volcanology, Industry

## Abstract

In this work, we present a comprehensive rheological database including most of the existing data relevant for crystal-bearing magmas collected from the scientific literature, covering the entire range of natural volcanic conditions, in terms of crystal content (1–80%), crystal shape (aspect ratio *R* from 1 to 13), and strain rate (between 10^−7^ and 10^2 ^s^−1^). Datasets were collected and discerned as a function of the information which we considered necessary for building a general systematic model describing relative viscosity of crystal-bearing magmas, such as the apparent and melt viscosity, the crystal concentration, crystal shape, and the strain rate. The selected dataset was then used for modelling the relative viscosity of a liquid-solid mixture having different concentrations of particles with different *R*, subjected to different strain rates. The proposed model allows us to quantitatively describe the rheological behaviour of crystal-bearing magmatic systems.

## Abstract (summary)

**Table:** 

Measurement(s)	Viscosity • Rheological Properties
Technology Type(s)	Viscometry
Factor Type(s)	Relative Viscosity
Sample Characteristic - Environment	Magma

## Background & Summary

In the last decades, understanding the rheological properties of materials has been a focal point in several fields, spanning from industry to geoscience. To date, different approaches and materials have been used, trying to describe the behaviour of different analogue systems.

Magma is a multiphase mixture made of silicate melt, crystals, and bubbles. As a function of the different magma composition, bubble and crystal contents and physical conditions (i.e. strain rate), its rheological response varies^1–6^. Due to the complexity of such a polyphase system, several open questions remain for an exhaustive comprehension of magma rheology. Through decades, scientists focused on the study of simplified systems, whose comprehension led to important steps forward towards the understanding of more complex systems. Here, we focus our attention on the rheology of two-phase particle-bearing mixtures of natural magmas, as a necessary building block for the comprehension of the behaviour of magmas in natural environments.

To date, multiple studies investigated the rheological behaviour of crystal-bearing silicate melts^1,5,7–34^.

**Fig. 1:**
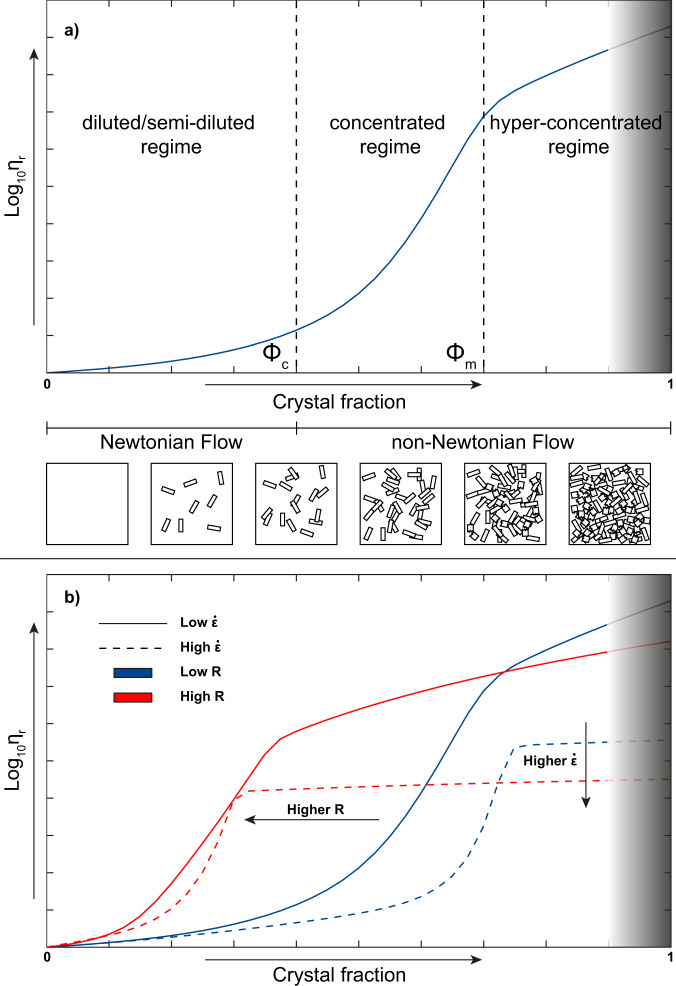
Relative viscosity-crystal fraction relationship for solid suspensions. (**a**) The figure shows the variation of relative viscosity and rheological regimes as a function of crystal fraction, highlighting a Newtonian (diluted and semi-diluted regimes) and a non-Newtonian behaviour (concentrated regime) beyond the critical particle fraction *ϕ*_*c*_, until a plateau-like viscosity region at high crystal fraction is attained above the maximum particle fraction *ϕ*_*m*_ (hyper-concentrated regime); modified after Lejeune and Richet (1995). (**b**) Effect of strain rate and aspect ratios of crystals on the sigmoidal shaped curves. The grey shaded area shows the range of crystal fraction in which the suspension has almost a solid behaviour and the deformational mechanisms change and strongly depend on the type of material, so it is not possible to estimate the relative viscosity based on the model assumptions made for this work.

In general, viscosity increases as the solid phase concentration of the mixture increases, according to a sigmoidal-shaped curve semi-qualitatively described for the first time by Lejeune and Richet (1995)^14^ and then through a mathematical parameterization by Costa^35^ and Costa *et al*.^1^ in terms of relative viscosity *η*_*r*_ (i.e., the value of measured apparent viscosity *η*_*a*_ divided by the viscosity of the suspending liquid, *η*_*l*_). From Fig. 1a, different flow regimes can be recognized^35–40^ (diluted, semi-diluted, concentrated and hyper-concentrated), describing a weak nonlinear variation of the mixture viscosity in the regime characterized by non-interacting suspended particles (diluted and semi-diluted regimes), till the achievement of a critical value (*ϕ*_*c*_), beyond which particles start to strongly interact among them, affecting considerably the increase of viscosity (concentrated regime), which is characterized by a strong non-linear variation and a significant yield stress^34^ (non-Newtonian behaviour). These three regimes depend not only on the concentration but also on the shape, size, distribution and orientation of crystals^34^. As the crystal fraction achieves its maximum packing (*ϕ*_*m*_), the mixture enters in the hyper-concentrated regime, and as the solid fraction further increases, deformational mechanisms change and strongly depend on the material properties of the solid phase.

A number of authors^39–48^ have focused their attention on the modelling of the viscosity of particle-bearing suspensions as function of the aspect ratios (*R*) of the suspended particles, especially in the diluted and semi-diluted regimes. However, most of these studies did not consider the concentrated regime and the effects of the strain rate on viscosity. It was demonstrated that, at high particle concentration, the transition between Newtonian and non-Newtonian behaviour occurs earlier as the strain rate increases^31,49–52^. At lower strain rates the behaviour is Newtonian and the increase in viscosity is mainly due to the textural features of the mixture itself^31,52,53^. As the strain rate overcomes a certain threshold, a decrease of apparent viscosity occurs, explained by a non-Newtonian fluid behaviour known as *shear-thinning*^54^, till the achievement of a minimum value. This value represents the onset of a pseudo-Bingham field, in which viscosity no longer depends on the strain rate. Based on that, many works have been carried out to improve the modelling of the rheological behaviour of particle-bearing mixtures.

A semi-empirical parameterization of relative viscosity valid for the whole range of particle concentration was proposed first by Costa^35^ and then modified and improved by Costa *et al*.^9^, and Costa *et al*.^1^ based on the experiments of Caricchi *et al*.^31^ and other available data^14,53,55–59^. This model is based on a four parameters equation that describes the variation of viscosity of particle mixtures as a function of the solid fraction at different strain rates:

1 $${\eta }_{r}=\frac{1+{\varphi }^{\delta }}{{[1-F(\varphi ,\varepsilon ,\gamma )]}^{B\phi * }}$$

where

2 $$F=\left(1-\xi \right){\rm{erf}}\left[\frac{\sqrt{\pi }}{2\left(1-\xi \right)}\varphi \left(1+{\varphi }^{\gamma }\right)\right]$$

with *B* being the Einstein coefficient (*B* = 2.5), *φ* = ϕ/ϕ*, and ϕ*, *ξ*, *γ* and *δ* empirical parameters which depend on the strain rate (Costa *et al*., 2009; Eq. 3^1^):

3 $$\begin{array}{ccc}{\phi }_{\ast }(\mathop{\varepsilon }\limits^{\cdot }) & = & {\phi }_{m}+\Delta \phi \frac{{(\mathop{\varepsilon }\limits^{\cdot }/{\varepsilon }_{c})}^{n}-{({\varepsilon }_{c}/\mathop{\varepsilon }\limits^{\cdot })}^{n}}{{(\mathop{\varepsilon }\limits^{\cdot }/{\varepsilon }_{c})}^{n}+{({\varepsilon }_{c}/\mathop{\varepsilon }\limits^{\cdot })}^{n}}\equiv {\phi }_{s}(\mathop{\varepsilon }\limits^{\cdot })\\ \delta (\mathop{\varepsilon }\limits^{\cdot }) & = & {\delta }_{m}-\Delta \delta \frac{{(\mathop{\varepsilon }\limits^{\cdot }/{\varepsilon }_{c})}^{n}-{({\varepsilon }_{c}/\mathop{\varepsilon }\limits^{\cdot })}^{n}}{{(\mathop{\varepsilon }\limits^{\cdot }/{\varepsilon }_{c})}^{n}+{({\varepsilon }_{c}/\mathop{\varepsilon }\limits^{\cdot })}^{n}}\equiv {\delta }_{s}(\mathop{\varepsilon }\limits^{\cdot })\\ \xi (\mathop{\varepsilon }\limits^{\cdot }) & = & {\xi }_{m}+\Delta \xi \frac{{(\mathop{\varepsilon }\limits^{\cdot }/{\varepsilon }_{c})}^{n}-{({\varepsilon }_{c}/\mathop{\varepsilon }\limits^{\cdot })}^{n}}{{(\mathop{\varepsilon }\limits^{\cdot }/{\varepsilon }_{c})}^{n}+{({\varepsilon }_{c}/\mathop{\varepsilon }\limits^{\cdot })}^{n}}\equiv {\xi }_{s}(\mathop{\varepsilon }\limits^{\cdot })\\ \gamma (\mathop{\varepsilon }\limits^{\cdot }) & = & {\gamma }_{m}+\Delta \gamma \frac{{(\mathop{\varepsilon }\limits^{\cdot }/{\varepsilon }_{c})}^{n}-{({\varepsilon }_{c}/\mathop{\varepsilon }\limits^{\cdot })}^{n}}{{(\mathop{\varepsilon }\limits^{\cdot }/{\varepsilon }_{c})}^{n}+{({\varepsilon }_{c}/\mathop{\varepsilon }\limits^{\cdot })}^{n}}\equiv {\gamma }_{s}(\mathop{\varepsilon }\limits^{\cdot })\end{array}$$

for which the values of the empirical parameters are reported in Table 1:

**Table 1:** Empirical parameters of Eq. 3, needed for the determination of the 4 constitutive parameters of the Eqs. 1 and 2.

n	ɛ_c_	ϕ_m_	Δ_ϕ_	δ_m_	Δ_δ_	ξ_m_	Δ_ξ_	γ_m_	Δ_γ_
0.30	4.07E-04	0.62	0.09	7.73	5.73	4.75E-04	4.56E-04	5.92	5.11

A step forward with respect to Costa *et al*.^1^ has been provided for the effects of the particle shape on relative viscosity by several authors^5,13,26,34^. They tried to quantify the control of particle aspect ratio on the rheological behaviour of magmas. However, except for Cimarelli *et al*.^34^, the proposed parameterizations approach to infinity as the crystal content tends to the hyper-concentrated regime, contrarily to the sigmoidal shaped model of Costa *et al*.^1^.

To summarize, rheological literature provides several rheological models, but none of them can provide a satisfactory fit of the behaviour of the mixture on the whole range of particle solid fractions accounting for the effects of strain rate and particle shape.

So, to overcome these shortcomings, we first built a general database of relative viscosity measurements for different applied strain rates and particle shapes. The database comprises more than 1400 rheological measurements for magmatic, synthetic, and analogue mixtures. Then we used the semi-empirical Eq. (1) of Costa *et al*.^1^ as starting point to build a more general model able to systematically describe the relationship relative viscosity – crystal content for variable strain rates and particle aspect ratios. The latter was possible by quantifying the dependence of the fitting parameters of Eqs. (1) and (2) with aspect ratio and strain rates, by using the novel rheological database built for this work.

## Methods

### Data analysis and classification criteria

The compilation of the database used to calibrate the numerical model includes over 1400 rheological data taken from over 40 scientific papers.

All data are classified as a function of the material type, distinguishing natural magma, synthetic melt, and analogue material datasets. In case of natural materials, the chemical composition is also reported. For all cases, the adopted experimental conditions were specified. The latter include temperature, applied strain rates, and confining pressure. Besides these, the *crystal fraction ϕ* together with the *mean aspect ratio R* were also provided. Results are presented in terms of *apparent viscosity η*_*a*_ of the mixture at the related *temperature T*, *melt viscosity η*_*l*_, and the *relative viscosity η*_*r*_.

Due to the large amount of collected data, an effective visual representation to discern one case from another was adopted. The entire database was subdivided into single bins associated to a value of *R* consisting of particles with aspect ratio between *R* - 0.5 and *R* + 0.5 at different intervals of strain rates spanning an order of magnitude each other. Indeed, all the considered strain rates used for our analyses were merged into discrete bins represented by the mid value of the strain rate bin spanning over one order of magnitude (which means that the variation from 10^−6 ^s^−1^ and 10^−5 ^s^−1^ is represented by the bin of 5 × 10^−6 ^s^−1^and so on). For the sake of clarity, we assigned a colour to each strain rate, and a symbol to each aspect ratio (see Supplementary Material “Details on the modelling strategy”).

### Limitations of the available datasets

It was not possible to use all the collected data for the parameterization. In fact, as we explain below, various shortcomings and limitations of part of the retrieved datasets did not allow their use:

1. Deformation mechanisms;
  The experimental methods commonly include 2 types of deformation, simple and pure shear deformation. As far as simple shear deformation is concerned, apparent viscosity is commonly reported in literature as $\sigma /\mathop{\varepsilon }\limits^{\cdot }$. Viscosity for pure shear deformation measurements (parallel plate deformation instruments) can be reported both simply as apparent viscosity ($\sigma /\mathop{\varepsilon }\limits^{\cdot }$) and as corrected following Gent^60^, which provides a correction of apparent viscosity for vertically deformed samples, taking into account the shape of the sample. Apparent viscosity is generally overestimated with respect to Gent’s, and it is difficult to properly correct the values if the geometrical parameters of the samples at the end of the deformation process are unknown.
2. Relative viscosity;
  In some datasets, *η*_*r*_ is not reported. This problem can be sometimes overcome, given the knowledge of the liquid viscosity. However, whereas for synthetic and analogue melts, it is sufficient to know the starting viscosity of the liquid phase, for natural melt the viscosity varies with time due to crystallization and differentiation of the residual melt. To properly calculate the relative viscosity, the residual liquid viscosity needs to be known at any time. This parameter is often missing, introducing an overestimation of the inferred relative viscosity, that cannot be used for this parameterization.
3. Missing information;

Some experimental measurements from the literature are unreliable because different physical processes occurring during the experiments are not properly constrained and therefore cannot be considered, such as viscous heating, strain partitioning or, simply, a not accurate calculation of the crystal fraction. In addition, for some experimental datasets, the required parameters for the fitting equation, such as the mean particle aspect ratio and /or even the applied strain rate are not reported^19,20,23–25^.

### New processing for incomplete datasets

From the compiled database we can clearly see that the available rheological measurements do not cover conditions wide enough to be fully representative of the range of natural volcanological settings.

In some cases, however, we were able to interpolate the existing measurements in order to complete the datasets:

- Picard *et al*.^15^ performed some of the experiments for *R* = 4 using the parallel plate techniques (pure shear deformation) and calculating the apparent viscosity as stress to strain rate ratio. This calculation gives an inherent overestimation of the viscosity as it does not consider the change in the shape of the specimen during deformation. For those experiments (samples with 38% of particles), a correction of about the 7% of the value was applied, after an experimental calibration^61^;

- Cimarelli *et al*.^34^ performed experiments using the concentric cylinder technique, at a constant particle amount, varying the strain rate by applying increasing and decreasing cycles. The value of viscosity of a sample deformed during an increasing strain rate cycle is higher than the value of viscosity of a sample deformed during a decreasing one, due to some deformational features^7,27^ (shear localization and combined effects of strain rate, strain and recovery of the strain among the different steps of the experiments): this generates a hysteresis cycle, with values of viscosity spanning over more than 2 orders of magnitude. Such variability increases as particle aspect ratio and solid fraction increase, reaching a maximum variability of 40% for the dataset corresponding to *R* = 9 and a volume fraction of 36%^34^. So, all the values were properly corrected, considering the upper limit in order to be consistent with the other data of the database;

- Ryerson *et al*.^28^ performed some of the experiments for *R* = 1 using the concentric cylinder techniques (vertical deformation), for particle concentrations from 0 to almost 70% and a strain rate up to 2 × 10^2^ s^−1^. Due to the high particle concentrations and strain rates, viscous heating may occur^62,63^, causing a wrong estimation of the viscosities. To overcome this problem, an estimation of the effect of viscous heating was performed, by estimating the Nahme number^62^ (see the Technical validation section) and the maximum increase of temperature (*∆T*) that may have affected the final value of viscosity. Once the *∆T* was estimated, we used the value of melt viscosity at the new temperature in order to obtain the correct value of relative viscosity.

With respect to Costa *et al*. (2009)^1^, we aim to systematically improve the approach by using more quantitative constraints and consistent data and reducing potential biases. For these reasons several datasets originally used by Costa *et al*. (2009)^1^ for the calibration of their model were excluded because they are not in line with the new used criteria or use experimental methods not consistent with the type of deformation considered in our study. In particular, different issues were recognized such as unspecified aspect ratios of the solid particles in the considered samples: this comprises the cases of Van der Molen and Paterson^58^, Auer *et al*.^64^, Rushmer^56^, Rutter and Neumann^55^, which were arbitrarily assumed as spherical particles by Costa *et al*. (2009)^1^. However, a correct parameterization requires the proper determination of the particle aspect ratio, so, these studies had to be excluded from the parameterization for lack of this information but, for the sake of comparison, we report the data in the Supplementary Fig. 8, showing how these data plot with respect the model predictions.

After the critical analysis of the existing literature, we were able to calibrate our numerical model with a selection of almost 800 rheological data from the bibliographic research.

### Reassessing the model of Costa *et al*. (2009) for spherical particles

The model of Costa *et al*.^1^ (2009^1^; Eqs. 1 and 2) comprises a 4 parameter (ϕ_*_, *ξ*, *γ*, and *δ*) semi-empirical equation, reproducing the functional sigmoidal curve described by Lejeune and Richet (settings 1995)^14^ for spherical particle-bearing mixtures.

Values of the 4 parameters for each strain rate (from 10^−7^ to 10^2 ^s^−1^) were obtained after the calibration with the dataset corresponding to *R* = 1.

Model fittings were performed by using Gnuplot software (http://www.gnuplot.info).

For spherical particles (i.e. with *R* = 1), the dependency of the 4 parameters of the rheological model (ϕ_*_, *ξ*, *γ* and *δ*; Eq. 1) with the strain rate ($\mathop{\varepsilon }\limits^{\cdot }$) is given by Eq. 3^1^.

**Fig. 2:**
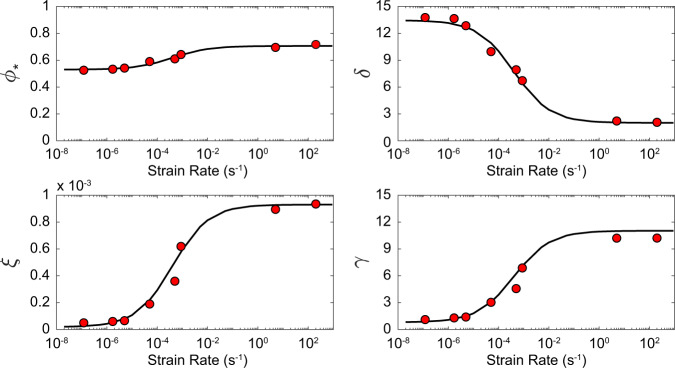
Strain rate dependence of parameters in Eq. 3 and Table 1 for spherical particles (*R* = 1).

The corresponding values of the constitutive parameters of Eqs. 1 and 2 for each strain rate (from 5 × 10^−7^ to 2 × 10^2 ^s^−1^), after the calibration with the new dataset for spherical particles and variable strain rates, are reported in Table 2. The performance of the fitting procedure for the parameters of Eq. 3 is shown in Fig. 2, together with the best-fit curves.

**Table 2:** Constitutive parameters of Eqs. 1 and 2 for spherical particle mixtures over the entire range of considered strain rates.

Strain-Rate (s^−1^)	5.00E-07	5.00E-06	5.00E-05	5.00E-04	5.00E-03	5.00E-02	5.00E-01	5.00E + 00	5.00E + 01	2.00E + 02
*δ*	13.26	12.70	10.93	7.38	4.08	2.61	2.16	2.04	2.01	2.00
*γ*	0.99	1.49	3.07	6.24	9.18	10.50	10.90	11.00	11.03	11.03
ϕ_*_	0.53	0.54	0.57	0.63	0.68	0.70	0.71	0.71	0.71	0.71
*ξ*	3.47E-05	7.93E-05	2.20E-04	5.03E-04	7.65E-04	8.82E-04	9.18E-04	9.27E-04	9.30E-04	9.30E-04

### Generalization of the model to different particle aspect ratios for the case of strain rate 5 × 10^−4 ^s^−1^

In order to extend the validity of the model to non-spherical particles (i.e. *R* > 1), we first considered the dataset corresponding to a strain rate of 5 × 10^−4 ^s^−1^, which is the most complete dataset covering different aspect ratios. In particular it includes 4 datasets corresponding to particle aspect ratios of *R* = 1^8,14,31,34^, *R* = 2^34^; *R* = 4^15^ and *R* = 9^34^.

The calibration of the model parameters on these datasets allowed us to evaluate the specific constitutive empirical parameters for the different values of *R*, which were used to obtain the trends of the 4 constitutive parameters as a function of the aspect ratio *R*.

Model fittings were performed by using Gnuplot software (http://www.gnuplot.info).

**Fig. 3:**
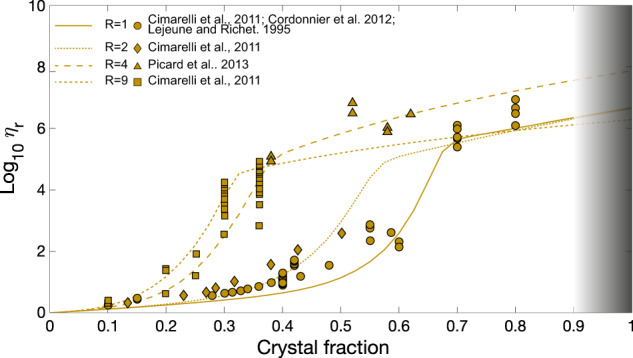
Fitting curves for datasets of *R* = 1,2,4 and 9, strain rate of 5 × 10^−4 ^s^−1^.

For this case, for each *R*, a fit of the parameters of Eqs. (1) and (2) was performed, as shown in Fig. 3, leading to the evaluation of the specific constitutive empirical parameters, reported in Table 3.

**Table 3:** Empirical fit of Eqs. (1) and (2) for the datasets having *R* of 1, 2, 4 and 9 at a strain rate of 5 × 10^−4 ^s^−1^.

R	ϕ_*_	*γ*	*δ*	*ξ*
1	0.62	7.38	6.24	5.03E-04
2	0.49	3.30	7.36	2.74E-04
4	0.26	1.38	6.81	1.00E-06
9	0.21	1.42	3.66	5.00E-08

**Fig. 4:**
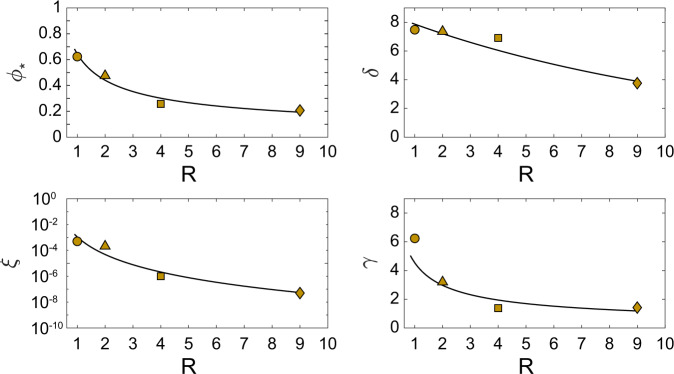
Trends of the empirical parameters as function of particle aspect ratios for the empirical parameters of the Eq. 4.

All the 4 empirical parameters of Eqs. (1) and (2) show decreasing trends as a function of *R* (Fig. 4 and Table 3), which have been modelled assuming power law and exponential relationships.

Hence, for $\mathop{\varepsilon }\limits^{\cdot }$ = 5 × 10^−4^ s^−1^ the dependency of the empirical parameters from *R* can be described as:

4 $$\begin{array}{lll}{\phi }_{* }\left(\mathop{\varepsilon }\limits^{\cdot }=1{0}^{-4}\;{s}^{-1},R\right) & = & \frac{1}{{R}^{{b}_{1}}}{\phi }_{s}\left(1{0}^{-4}\;{s}^{-1}\right)\\ \delta \left(\mathop{\varepsilon }\limits^{\cdot }=1{0}^{-4}\;{s}^{-1},R\right) & = & {\delta }_{m}{e}^{{b}_{2}(R-1)}\left(1{0}^{-4}\;{s}^{-1}\right)\\ \xi \left(\mathop{\varepsilon }\limits^{\cdot }=1{0}^{-4}\;{s}^{-1},R\right) & = & \frac{1}{{R}^{{b}_{3}}}{\xi }_{m}\left(1{0}^{-4}\;{s}^{-1}\right)\\ \gamma \left(\mathop{\varepsilon }\limits^{\cdot }=1{0}^{-4}\;{s}^{-1},R\right) & = & \frac{1}{{R}^{{b}_{4}}}{\gamma }_{m}\left(1{0}^{-4}\;{s}^{-1}\right)\end{array}$$

where *b*_1_ = 0.538, *b*_2_=0.092, *b*_3_ = 4.568 and *b*_4_ = 0.718, and *ϕ*_s_, *ξ*_*m*_, *γ*_*m*_, and *δ*_*m*_ are the values meant for the spherical particles at $\mathop{\varepsilon }\limits^{\cdot }$ = 5 *×* 10^−4^) s^−1^.

### Final equation model for particle-bearing systems

The relationship of the parameters as function of the aspect ratios found for $\mathop{\varepsilon }\limits^{\cdot }$ = 5 × 10^−4^ s^−1^ was assumed to be valid for all the other strain rates and used for a more general parameterization valid for the entire range of strain rate and aspect ratios of volcanic interest.

Model fittings were performed by using Gnuplot software (http://www.gnuplot.info).

The relationships of Eq. 4 describing the effects of particle aspect ratios on relative viscosity for the case at $\mathop{\varepsilon }\limits^{\cdot }$ = 5 × 10^−4^ s^−1^ were used to build a general parameterization:

5 $${\eta }_{r}(\mathop{\varepsilon }\limits^{\cdot },R)=\frac{1+{\varphi }^{\delta (\mathop{\varepsilon }\limits^{\cdot },R)}}{{[1-F(\mathop{\varepsilon }\limits^{\cdot },R)]}^{B{\phi }_{* }(\mathop{\varepsilon }\limits^{\cdot },R)}}$$

valid for the entire range of strain rate and aspect ratios, by considering the relationships in Eqs. (3) and (4):

6 $$\begin{array}{lll}{\phi }_{\ast }(\mathop{\varepsilon }\limits^{\cdot },R) & = & \left[{\phi }_{m}+\Delta \phi \frac{{\left(\mathop{\varepsilon }\limits^{\cdot }/{\varepsilon }_{c}\right)}^{n}-{\left({\varepsilon }_{c}/\mathop{\varepsilon }\limits^{\cdot }\right)}^{n}}{{\left(\mathop{\varepsilon }\limits^{\cdot }/{\varepsilon }_{c}\right)}^{n}+{\left({\varepsilon }_{c}/\mathop{\varepsilon }\limits^{\cdot }\right)}^{n}}\right]\ast {R}^{-{b}_{1}}\\ \delta (\mathop{\varepsilon }\limits^{\cdot },R) & = & \left[{\delta }_{m}-\Delta \delta \frac{{\left(\mathop{\varepsilon }\limits^{\cdot }/{\varepsilon }_{c}\right)}^{n}-{\left({\varepsilon }_{c}/\mathop{\varepsilon }\limits^{\cdot }\right)}^{n}}{{\left(\mathop{\varepsilon }\limits^{\cdot }/{\varepsilon }_{c}\right)}^{n}+{\left({\varepsilon }_{c}/\mathop{\varepsilon }\limits^{\cdot }\right)}^{n}}\right]\ast {e}^{-{b}_{2}(R-1)}\\ \xi (\mathop{\varepsilon }\limits^{\cdot },R) & = & \left[{\xi }_{m}+\Delta \xi \frac{{\left(\mathop{\varepsilon }\limits^{\cdot }/{\varepsilon }_{c}\right)}^{n}-{\left({\varepsilon }_{c}/\mathop{\varepsilon }\limits^{\cdot }\right)}^{n}}{{\left(\mathop{\varepsilon }\limits^{\cdot }/{\varepsilon }_{c}\right)}^{n}+{\left({\varepsilon }_{c}/\mathop{\varepsilon }\limits^{\cdot }\right)}^{n}}\right]\ast {R}^{-{b}_{3}}\\ \gamma (\mathop{\varepsilon }\limits^{\cdot },R) & = & \left[{\gamma }_{m}+\Delta \gamma \frac{{\left(\mathop{\varepsilon }\limits^{\cdot }/{\varepsilon }_{c}\right)}^{n}-{\left({\varepsilon }_{c}/\mathop{\varepsilon }\limits^{\cdot }\right)}^{n}}{{\left(\mathop{\varepsilon }\limits^{\cdot }/{\varepsilon }_{c}\right)}^{n}+{\left({\varepsilon }_{c}/\mathop{\varepsilon }\limits^{\cdot }\right)}^{n}}\right]\ast {R}^{-{b}_{4}}\end{array}$$

with *b*_1_, *b*_2_, *b*_3_ and *b*_4_ defined by the Eq. 4.

The complete database (file ‘*Database_ScientificData.xlsx’*) and the final parameterization (file *‘Parameterization_ScientificData.xlsx’*) are available in Excel format, uploaded on figshare repository^65^ 10.6084/m9.figshare.16886155.v1. A full description of the dataset is reported in the file *‘Instructions.txt’*, available at the same link. Model fittings (Eqs. 5 and 6) were performed by using Gnuplot software (http://www.gnuplot.info).

## Data Records

### Compilation of the rheological database

The database consists of relative viscosity data obtained from the analysis of natural, synthetic, and analogue samples, covering a wide range of crystal content (1–80%) and different strain rates (between 10^−7^ and 10^2 ^s^−1^), typical of volcanic activity, and particle aspect ratios from 1 to 13, the range of which includes the large shape variability of the solid components within magma, from leucites (spherical) to plagioclases and feldspars, having variable aspect ratios. This shape variability, at the same strain rate and chemical composition, determines a consistent change in the rheological response of the materials^3,40^.

The database is reported as an excel file consisting of 3 different sheets, to distinguish natural, synthetic, and analogue materials. Each sheet reports all the main experimental features of the cited studies, in columns. These features consist of: article (citation), material, composition and source of the samples, experimental method, temperature (T °C), crystal fraction (*Ф*), mean aspect ratio (*R*), Stress (Pa), strain rate (s^−1^), Log melt viscosity (Pa s), Log apparent viscosity (Pa s), Log relative viscosity, Accuracy. An additional sheet is included, reporting additional datasets not suitable for the parameterization for lack of details.

The complete magma rheology database based on literature studies has been reported in an Excel file named “Database_ScientificData.xlsx”, uploaded on figshare repository^65^ 10.6084/m9.figshare.16886155.v1. The presented rheological model implemented as an Excel file named “Parameterization_ScientificData.xlsx” is also available. In addition, a text file with the instruction of the database named “Instructions.txt” is published.

## Technical Validation

### Testing the model on the complete database

**Fig. 5:**
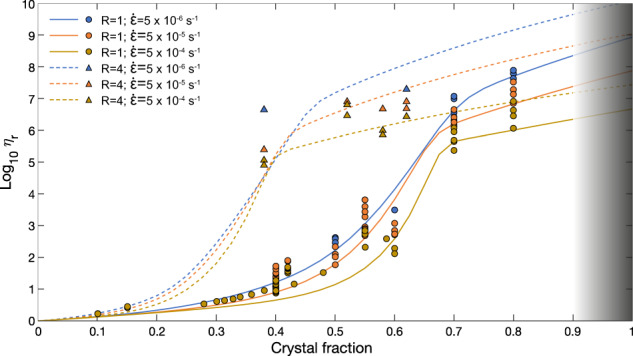
Plots for two different particle aspect ratios *R* and strain rates ranging from 5 × 10^−6^ to 5 × 10^−4 ^s^−1^.

Once the parameters controlling the particle shape effects were estimated for the case $\mathop{\varepsilon }\limits^{\cdot }$ = 5 × 10^−4^ s^−1^ and the model generalized, the performance of the latter was tested on all the other cases for which there are less available data, preventing to use them for quantitative fittings, as reported in the Supplementary Material (Details on the modelling strategy). As an example, the cases for *R* = 1 and *R* = 4 for different strain rates are reported in Fig. 5. In particular, Fig. 5 shows the application of the model to 3 strain rates (5 × 10^−6^, 5 × 10^−5^, and 5 × 10^−4^ s^−1^). For these cases, we have the possibility to compare the results both in the diluted and in the concentrated regime, which represents a very important novelty, as data covering the entire range of crystal fractions are typically quite scarce in the literature.

As we mentioned above, the available data for different strain rates do not cover the entire range of crystallinity. High particle fraction datasets are populated only for spherical particles^31,32^ and for *R* = 4^15^. However, taking into proper consideration the data scatter and uncertainties, the model is in good agreement with the data.

Concerning the high crystallinity regime, data, as shown in Fig. 5 (and the Supplementary Figures in the Supplementary material “Details on the modelling strategy”), shows a variability of relative viscosity of about one order of magnitude, even for very similar values of *R* and strain rate. Such intrinsic variability at high solid fractions must be always taken into consideration when performing rheological analysis, as it may be due to crystal breaking (and consequent particle polydispersity), hysteresis processes, and different deformational features occurring during the measurements, as also reported by Cimarelli *et al*.^34^ and described in the Supplementary material (Details on the modelling strategy).

As already predicted by the model of Costa *et al*.^1^ and applied to the dataset of Caricchi *et al*.^31^, a high crystal fraction plateau-like relative viscosity region (where there is a significant decrease of the slope of the relative viscosity) is recognizable for each dataset. For a given *R*, this plateau-like region is higher as the strain rate decreases. This is evident in Fig. 5 where blue dots (*R* = 1 and strain rate of 5 × 10^−6^ s^−1^) show higher relative viscosity values, with respect to the orange dots (*R* = 1 and strain rate of 5 × 10^−5^ s^−1^), which lay in the middle, and even more with respect to the yellow dots (*R* = 1 and strain rate of 5 × 10^−4^ s^−1^). Such a plateau-like region is also clearly recognizable for the dataset corresponding to *R* = 4^15^ (and other measurements made in this regime showed in the Supplementary material “Details on the modelling strategy”) shown in Fig. 5, although it is achieved at much lower crystal fractions with respect to the spherical particles. In general, at a given strain rate, the sigmoidal shaped curves of the model are systematically shifted towards the lower crystal fractions as *R* increases. Although data are lacking for the diluted and semi-diluted regimes, its lack of data is not very critical as the model agrees with the datasets at lower solid fractions available for the other values of *R* and strain rates (see “Details on the modelling strategy” in the Supplementary material).

An inference that clearly emerges from the analysis of the rheological database is that literature data of relative viscosity for solid fractions higher than 60%^15^ and for aspect ratios larger than 1 are scarce or entirely missing with respect to the dataset for spherical particles for which data are available up to 80% of crystal fraction. Therefore, it is not possible for us to compare the trends of the model for *R* = 4^15^ for crystal fraction higher than 60%, at the same strain rate. Further work is necessary and underway to fill this gap.

The grey shaded area starting close to the melt connectivity transition (MCT), at about 90% of crystal fraction, corresponds to the region above the percolation threshold beyond which the solid matrix inhibits liquid interconnectivity^1^. Deformation mechanisms change in this region, so rheological results cannot be merely obtained by an extrapolation of the model.

Summarizing, the model is reliable for the aspect ratios and strain rate used for the calibration (*R* 1, 2, 4 and 9 and $\mathop{\varepsilon }\limits^{\cdot }$ from 10^−7^ to 10^2^ s^−1^) and has an uncertainty of ±1 log unit. Tests made for other aspect ratios and strain rates, that were not used in the calibration, show that the data are also in agreement with the model.

**Fig. 6:**
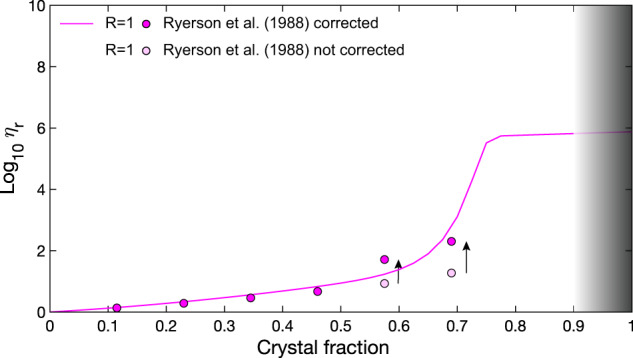
Dataset from Ryerson *et al*. (1988) showing viscous heating effects for high deformational strain rates and high particle concentration, corrected according to the procedure proposed by Costa *et al*. (2007).

A very significant case of a natural sample (silicate melt and crystals) deformed at a very high strain rate (up to 2 × 10^2^ s^−1^) was presented by Ryerson *et al*.^28^. The authors observed a low relative viscosity increase with an increasing solid fraction in the concentrated regime and, as mentioned by the same authors, this could be due to an overestimation of the local magma viscosity due to viscous heating effects^62,63^. As shown in Fig. 6, the model overshoots the original dataset of Ryerson *et al*. (1988)^28^. Indeed, effects of viscous heating, during the experiments at high strain rates, are larger for higher crystal content^51–53^, increasing the local temperature and hence decreasing the liquid viscosity, so increasing the relative viscosity *η*_*r*_.

In order to correct the effect of this local temperature increase, responsible for the underestimation of the apparent viscosity, data related to the two data at the highest crystal fractions were corrected following the method described by Costa *et al*. (2007)^62^. To do this, we first needed to estimate the Nahme number *Na*:

7 $$Na=\frac{b{\eta }_{a}{V}^{2}}{k}$$

where *η*_*a*_ is the apparent viscosity value, *b* is the rheological sensitivity to the temperature (1/*η*_*a*_ d*η*_*a*_/dT), *V* is the velocity of deformation, and *k* is the thermal conductivity. Knowing the viscosity in the Arrhenius form in terms of activation energy *E*_*a*_, *b* can be estimated as *b* = *E*_*a*_/*T*_0_^2^) (with *T*_0_ reference absolute temperature of the considered range^66–68^), while *V* can be obtained by multiplying the reported strain rate of deformation (2 × 10^2^ s^−1^) with the thickness of the sample (reported as 0.00825 m).

As explained by Costa *et al*.^62^, viscous heating effects considerably affects the viscosity and flow dynamics when *Na* ≫ 1. From Eq. (7)^62^, we can estimate the maximum variation of temperature as:

8 $$\Delta T=\frac{{\rm{In}}\left(Na\right)}{b}$$

Table 4 reports the values of Nahme number and the related maximum variation of *T* occurred for the two highest crystal fractions of the dataset of Ryerson *et al*. (1988)^28^.

**Table 4:** Parameters for the calculation of the Nahme number for the correction of the viscous heating effects for the viscosity at crystal fractions of 57% and 69%.

*ϕ*	*η*_a_	b	k	V	Na	Δ*T*
0.12	57.80	0.01	1	1.65	2.29	—
0.23	112.91	0.01	1	1.65	4.48	—
0.35	236.03	0.01	1	1.65	9.37	—
0.46	540.12	0.01	1	1.65	21.44	—
0.58	1405.91	0.01	1	1.65	55.81	136
0.69	4485.98	0.01	1	1.65	178.07	178

Considering the values reported by Ryerson *et al*.^28^, the corresponding values of *Na* in the concentrated regime are much larger than one (see Table 4), so the related variation of temperature can be responsible of a significant overestimation of the melt viscosity. This temperature variation is here estimated, respectively, as 178 °C and 136 °C for 69% and 58% of crystal content. These values were then used for the correction of the apparent viscosity^28^. In Fig. 6, relative viscosity data corrected for the maximum estimated variation of temperature are reported, resulting in a good agreement with the model.

To summarize, for the comprehension of the effects of strain rate, concentration and shape of crystals on the rheology of natural silicate mixtures, there is the need of a large amount of data, covering at least the most relevant cases. This need led to the compilation of a rheological database, through the collection of data from 40 scientific rheological papers. Such a compilation was then used for the generalization of the equation proposed by Costa *et al*., 2009^1^. The parameters of the model were originally suitable only for spherical particles and for strain rates ranging from 10^−6^ to 10^−3 ^s^−1^. However, the collected data allowed us to extend the validity of the model to a wider range of strain rates, including all the deformation conditions in nature. Moreover, we were also able to obtain a general empirical model to systematically predict the relative viscosity of mixtures having particles with aspect ratios much larger than 1. An accurate data analysis enabled to identify deformational features and problems affecting the results of the measurements reported in the papers. On this basis, despite the huge amount of data, a significant number of datasets were discarded.

At the present state, the model proposed here performs very well within the datasets used for the calibration^8,14,15,31,34^ and, with an uncertainty of 1 log units, the results can be extended to the other aspect ratios and strain rates existing in nature. By carefully analysing all the literature, we were able to correctly identify gaps in the multiparameter space which could be investigated, in the near future, by the scientific community to further test the validity and refine the presented model.

## Supplementary information

A comprehensive database of crystal-bearing magmas for the calibration of a rheological model

## Data Availability

The complete database of the rheological data based on literature studies has been reported in an Excel file, uploaded on figshare repository^65^ . The presented rheological model has been implemented in an Excel file, uploaded on figshare repository^65^ 10.6084/m9.figshare.16886155.v1.
