# Supplementary material for: A comprehensive database of crystal-bearing magmas for the calibration of a rheological model
Source: Sci Data. 2022 May 30;9:247. doi: 10.1038/s41597-022-01363-w (PMC9151800; doi:10.1038/s41597-022-01363-w)
Supplement: Supplementary file 1 — A comprehensive database of crystal-bearing magmas for the calibration of a rheological model [file 41597_2022_1363_MOESM1_ESM.pdf]

Scientific Data

Supplementary material for

A comprehensive database of crystal-bearing magmas for the calibration of a rheological model

Alessandro Frontoni[1,*], Antonio Costa[2], Alessandro Vona[1], Claudia Romano[1]

[1]Dipartimento di Scienze sez. Geologia, Università degli Studi Roma Tre, L.go San Leonardo Murialdo 1, 00146 Rome, Italy

[2]Istituto Nazionale di Geofisica e Vulcanologia, Sezione di Bologna, Via Donato Creti, 12, 40128 Bologna, Italy

*Corresponding author: Alessandro Frontoni (alessandro.frontoni@uniroma3.it)

Table of contents

Strain rate and aspect ratio database classification in discrete bins (including Supplementary figure 1)

Model application and comparison with the available datasets (including Supplementary figure 2 to Supplementary figure 8)

Strain rate and aspect ratio database classification in discrete bins

Due to the large amount of data collected for the rheological database, an effective visual representation to discern one case from another was adopted. For the sake of clarity, we assigned a different colour to each strain rate bin, and a different symbol to each aspect ratio bin, as summarized in Supplementary figure 1 below. Each strain rate bin, represented by the mid interval value, spans over an order of magnitude. So, for instance, the bin represented by 5×10^{-6} s^{-1}, encompasses all the data obtained under strain rates between 10^{-6} s^{-1} and 10^{-5} s^{-1}, and so on.

Similarly, each aspect ratio bin covers all cases ranging from R-0.5 to R+0.5. So, for instance, the bin represented by R=2, includes all the data relative to particles having aspect ratios between 1.5 and 2.5, and so on.

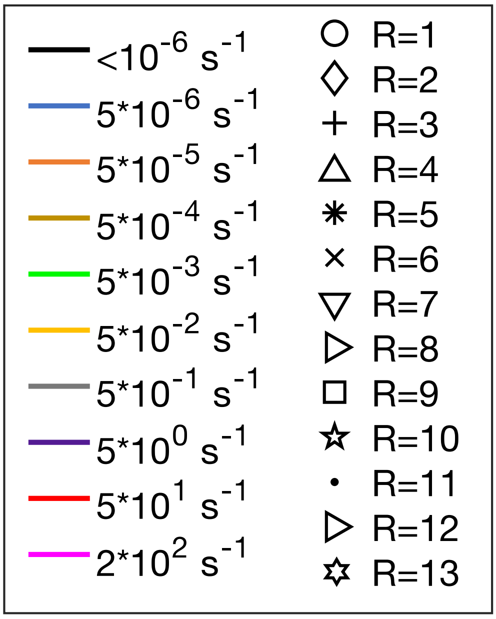

Supplementary figure 1- Chromatic and symbolic legend standing for, respectively, different strain rate and aspect ratio bins used to subdivide the entire dataset.

Model application and comparison with the available datasets

The model was applied to all the available dataset having different *R* and strain rates.

As for the case of strain rate 5 × 10^{-4} s^{-1}, described in the main text, the available datasets do not cover the entire range of crystallinity.

Starting from the lower strain rates, the dataset at 5 × 10^{-6} s^{-1} is populated by the data for *R*=1[26], which were already discussed in the main text, and for *R*=4[10], for the concentrated regime (Supplementary figure 2). This dataset is missing relative viscosity measurements in the diluted and semi-diluted regimes. However, the few available data are in good agreement with the general trend of the model.

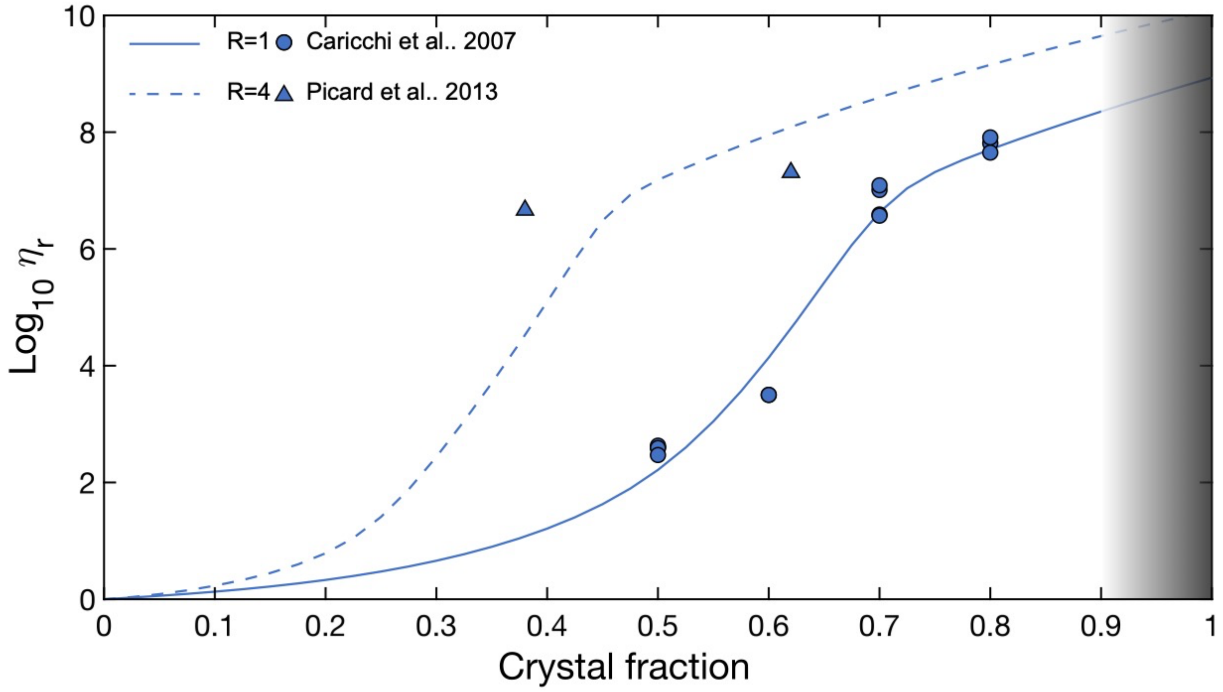

Supplementary figure 2 - Dataset and model comparison for strain rates of 5 × 10^{-6} s^{-1} and different *R*.

The dataset at strain rate of 5 × 10^{-5} s^{-1} and different *R* (Supplementary figure 3), consists of measurements for spherical particles[8,26,30] (*R*=1), and for *R*=4[10]. Even in this case, available data are in good agreement with the model prediction although data for the diluted and semi-diluted regimes are missing. Although the model seems to underestimate the relative viscosities for the case of the spherical particles, we need to highlight that data labelled here as spherical

include particles with an aspect ratio between *R*-0.5 and *R*+0.5, and the data with 40% and 53% crystallinity have *R* = 1.4[30] and therefore stand correctly above the curve corresponding to *R*=1.

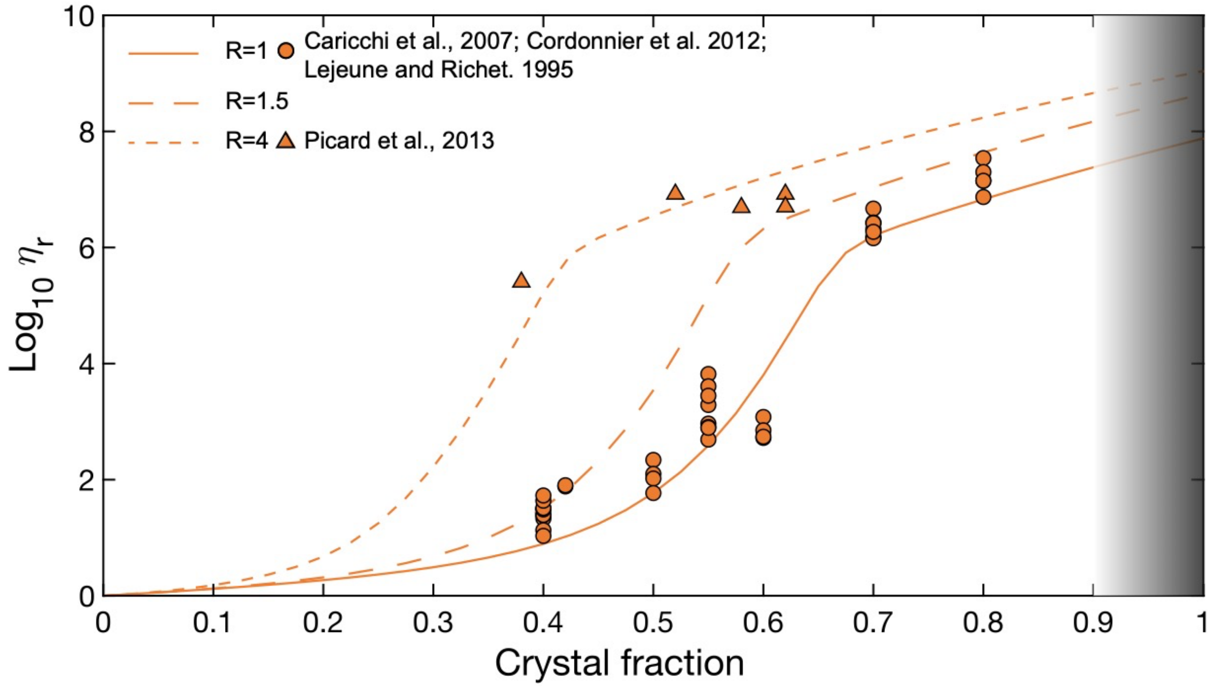

Supplementary figure 3 - Dataset and model comparison for strain rates of 5×10^{-5} s^{-1} and different *R*. Data with *R*=1 comprehend particle with aspect ratios ranging between *R*=1 and *R*=1.4 and stand exactly between the two curves of the model representing *R*=1 and *R*=1.5.

The dataset relative to strain rate of 5×10^{-3} s^{-1} and different *R* (Supplementary figure 4), is less represented, and consists of two measurements only for *R*=4[10], which appear to be in good agreement with the model, and data for spherical particles[8,9]. Data reported in the Supplementary Figure 4 appear to agree with the model prediction in the diluted regimes although the model seems to underestimate the relative viscosities as the mixture as the mixture approaches the concentrated regime at 53 % of particles.

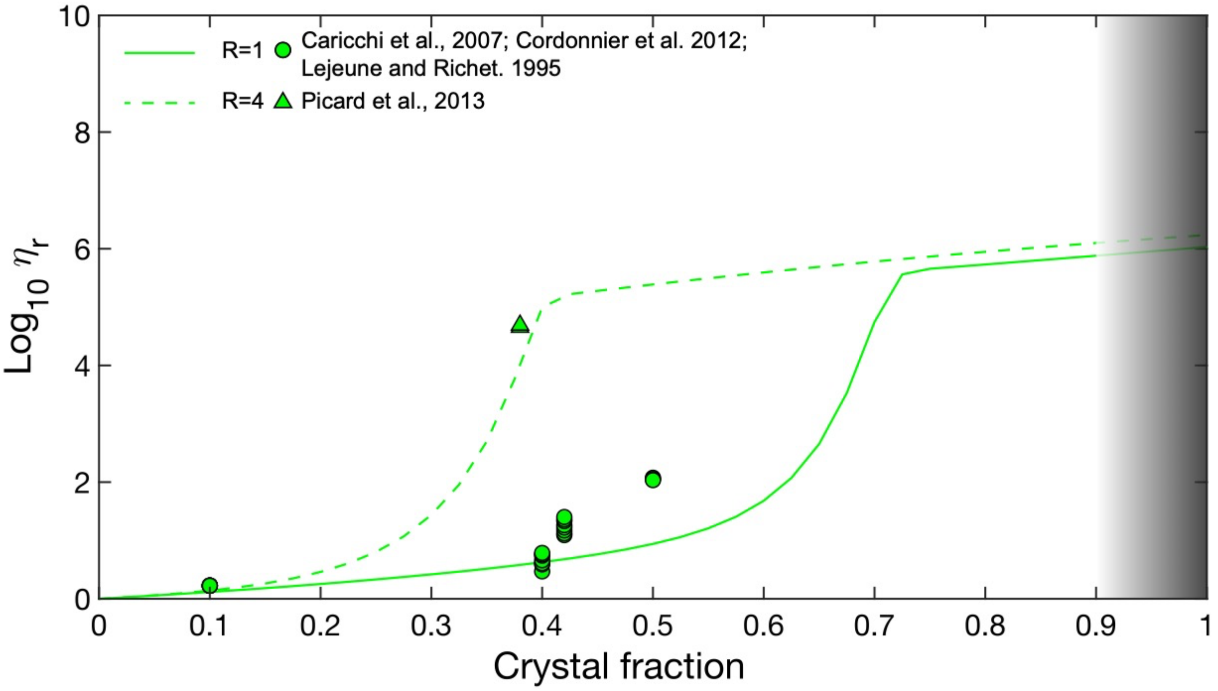

Supplementary figure 4 - Dataset and model comparison for strain rates of 5×10^{-3} s^{-1} and different *R*.

Concerning the dataset relative to strain rate of 5×10^{-2} s^{-1} (Supplementary figure 5), available data are also consistent with the model prediction, especially for *R*=9[28] and 13[66], whereas the data for *R*=1[8,9] do not follow the trend predicted by the model.

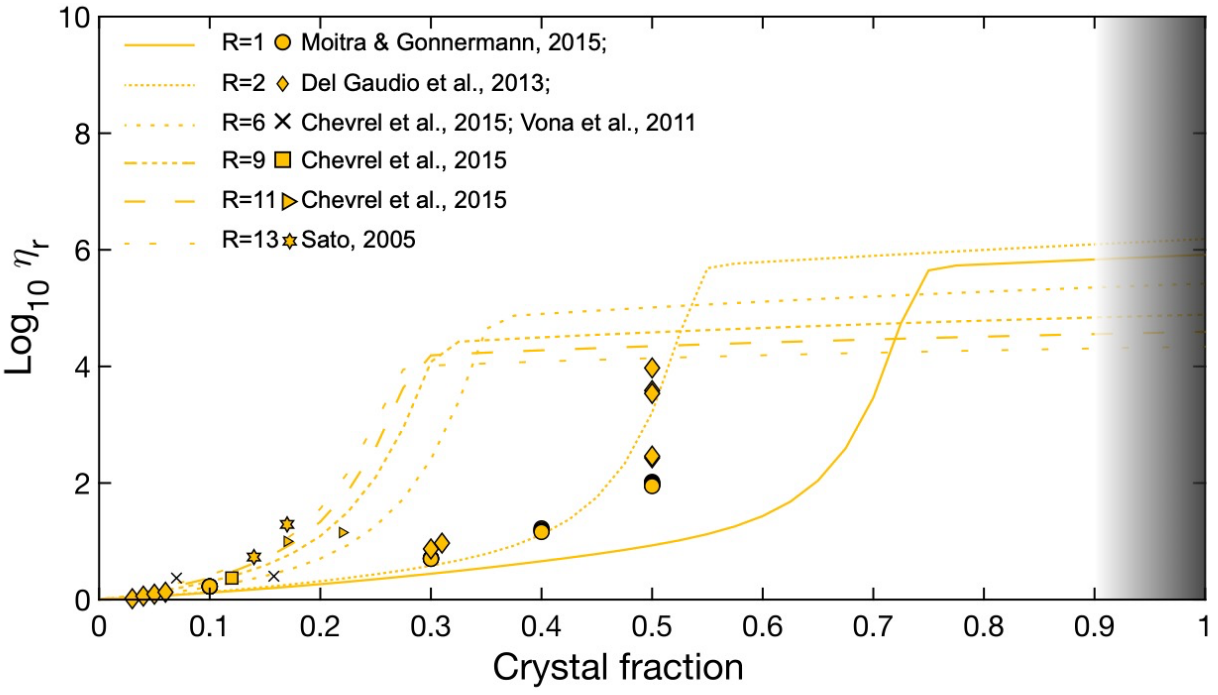

Supplementary figure 5 - Dataset and model comparison for strain rates of 5×10^{-2} s^{-1} and different *R*.

The dataset corresponding to strain rate of 5×10^{-1} s^{-1} presents a wide range of aspect ratios (Supplementary figure 6).

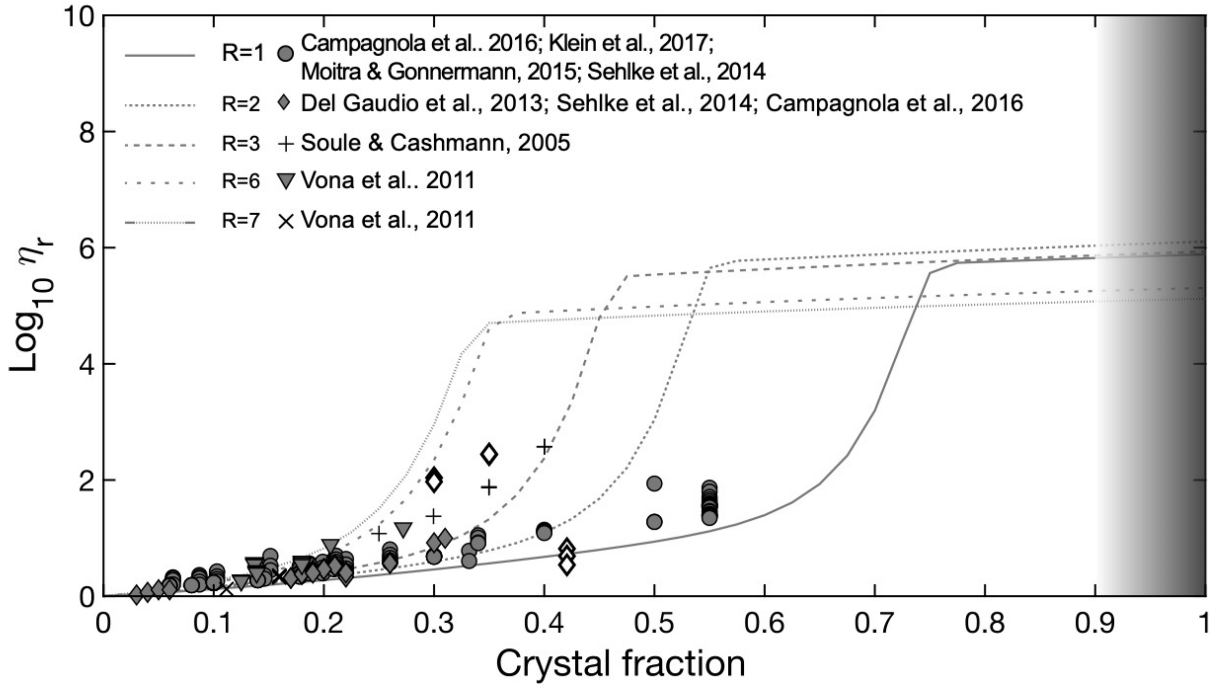

Supplementary figure 6 - Dataset and model comparison for strain rates of 5×10^{-1} s^{-1} and different *R*. Empty data corresponds to the measurements for *R*=2 from Del Gaudio et al. (2013) and Sehlke et al. (2014), deviating from the general trend.

In this case the trend for spherical particles data[7,9,11,24] is in agreement with the model but with a small systematic bias toward higher values with respect to the model predictions. For *R*=2[4,11,24], the data are generally in good agreement with the model, with few exceptions: 3 measurements of Del Gaudio et al., 2013[4] display relative viscosity values much higher than those predicted by the model. Despite apparently obtained through the same experimental conditions, those data points stand outside the general trend, maybe due to the occurrence of some deformational mechanisms affecting the viscosity. So, we considered these three data as outliers and discarded from further considerations. Concerning the data from Sehlke et al. (2014)[11], they all show different relative viscosities compared to those of the model with the exception of the data belonging to the diluted regime. An explanation of this may be the insurgence of viscous heating, not considered by the authors, occurring at high crystallinity and high strain rate. For *R*=3[17], the dataset shows a slight deviation from the model. The liquid viscosity η_l (reported in the paper) adopted for the calculation of the relative viscosity is an

averaged value, due to an imprecise evaluation of the dwell temperature (±25 °C). Concerning the case of R=6 and 7[22], the dataset is in good agreement with the model up to the concentrated regime, where there appear to be a slight deviation from the model curves. We do not have at the moment a clear explanation for this deviation.

The dataset corresponding to strain rate of 5×10^0 s^{-1} also spans over a wide range of R (Supplementary figure 7). As for the case of strain rate 5×10^{-1} s^{-1}, data for R=3[17] are quite in a good agreement, though the η_r is estimated using a poorly constrained η_l. The dataset for R=5[15] deviates from the model as the crystallinity approaches the concentrated regime (at a 20% of crystallinity for R=5, in red). Also in this case, as the mixture approaches the concentrated regime and the strain rate increases, the results of the experiments might be influenced by viscous heating or shear localization, not considered in the paper, but reasonable, due to the conditions of the experiments.

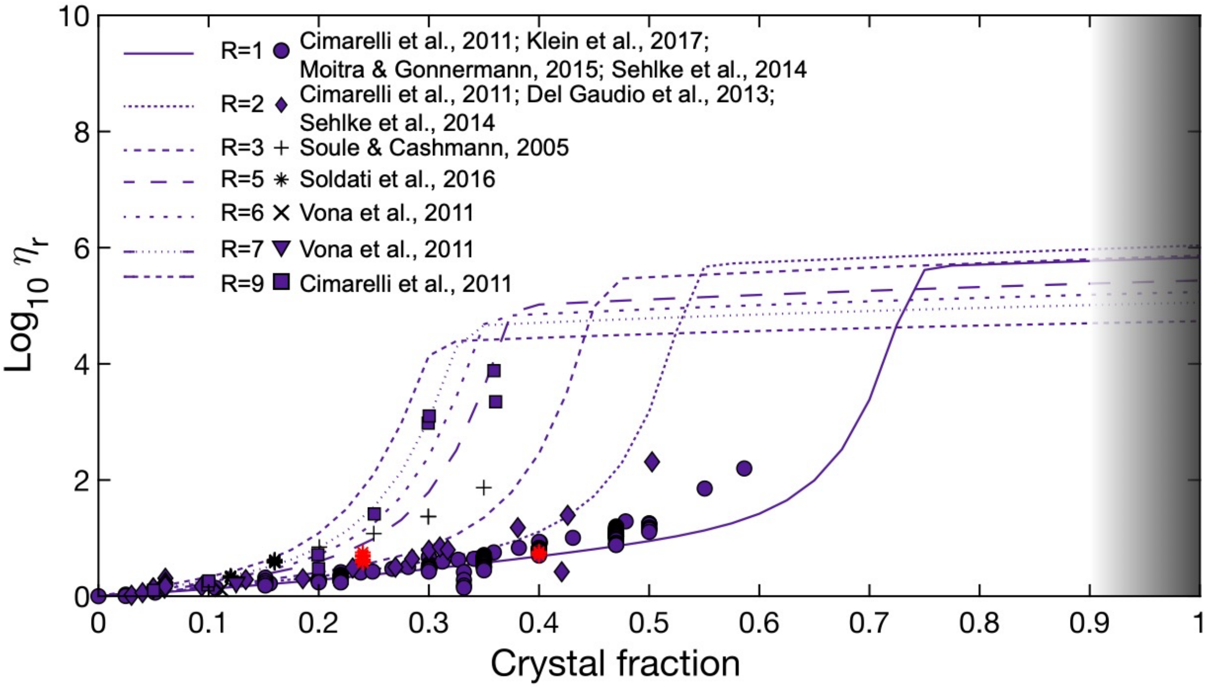

Supplementary figure 7 - Case for different R and strain rate of 5×10^0 s^{-1}.

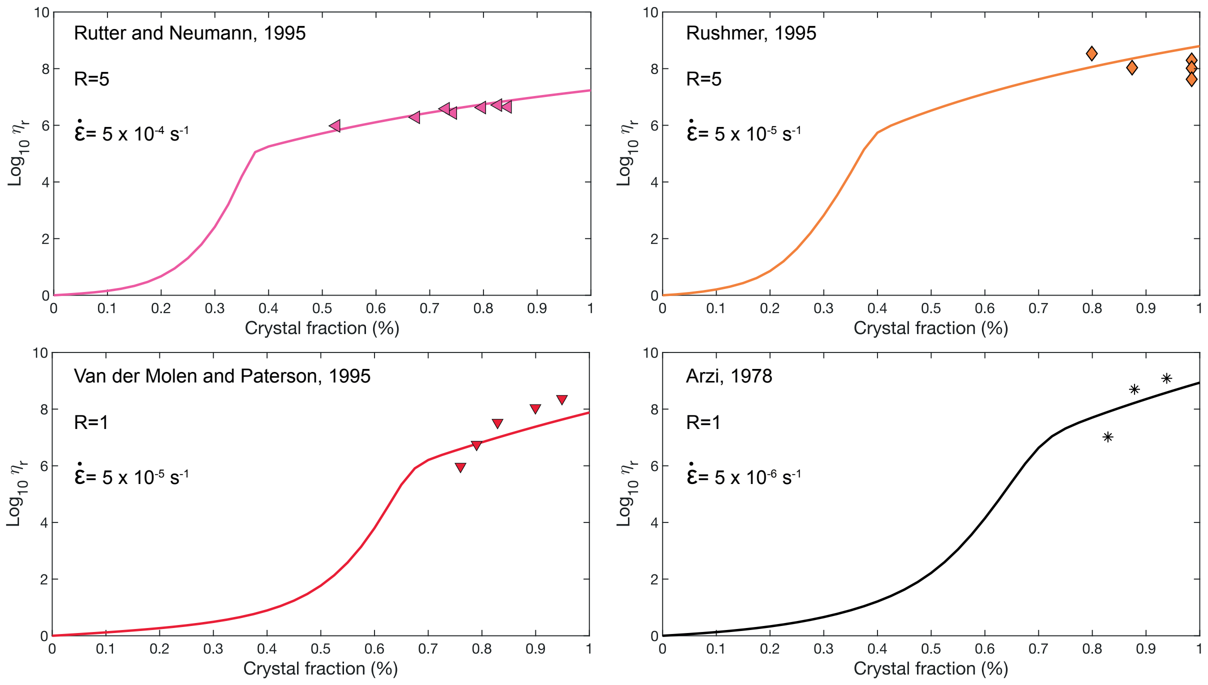

Supplementary figure 8 - Additional literature datasets used in Costa et al. (2009) but not used for constraining the new parameterization because of missing information. Data are compared with reasonable fitting curves for comparison purposes only.

In conclusion, the model can be considered reliable for the aspect ratios for which the calibration was performed at a strain rate 5×10^{-4} s^{-1}. The parameterization can also be extended with a certain accuracy to the other aspect ratios and strain rates typical of natural environments, although the paucity of data prevents to use them for a more robust calibration of the model. To optimize and refine the parameterization in all the parameter space, we replicate the urgency to provide new well-constrained measurements especially at high strain rates (higher than 10^{-3} s^{-1}) and R (higher than 3) in the concentrate regime (ϕ larger than 50%).
